# 基于416例Ⅰ期NSCLC肺叶切除术后随访结果探讨早期NSCLC术后随访策略

**DOI:** 10.3779/j.issn.1009-3419.2018.03.15

**Published:** 2018-03-20

**Authors:** 亮 戴, 万璞 闫, 晓征 康, 浩 付, 永波 杨, 海涛 周, 震 梁, 宏超 熊, 瑶 林, 克能 陈

**Affiliations:** 100142 北京，北京大学肿瘤医院暨恶性肿瘤发病机制及转化研究教育部重点实验室，胸外一科 Key Laboratory of Carcinogenesis and Translational Research (Ministry of Education), the First Department of Thoracic Surgery, Peking University Cancer Hospital and Institute, Peking University School of Oncology, Beijing 100142, China

**Keywords:** 肺肿瘤, 肺叶切除术, 预后, 随访, Lung neoplasms, Lobectomy, Prognosis, Follow-up

## Abstract

**背景与目的:**

目前国际上对于早期非小细胞肺癌（non-small cell lung cancer, NSCLC）的随访策略（随访间隔时间和随访内容）并未达成共识，相关的临床证据也十分有限。本研究通过Ⅰ期NSCLC的随访结果，总结其复发转移的部位及时间，为制定该类患者的随访间隔时间和内容提供参考。

**方法:**

回顾性分析我科肺癌前瞻性数据库中2000年1月-2013年10月单一医生组连续行解剖性肺叶切除手术的416例Ⅰ期NSCLC患者，根据复发转移部位及时间，探讨该类患者的长期随访间隔时间和内容。

**结果:**

全组患者5年无疾病生存率（disease free survival, DFS）与总生存率（overall survival, OS）分别为82.4%和85.4%；随访期间出现复发转移者共76例（18.3%），复发转移部位中常见者依次为肺转移21例（5.0%）、脑转移20例（4.8%）、骨转移12例（2.9%）和纵隔淋巴结转移12例（2.9%）。影响复发转移的因素中，pT2a者复发转移率高于pT1者（*P*=0.006），5年DFS分别为73.8%和87.6%（*P*=0.002），5年OS分别为77.7%和90.3%（*P*=0.011）。

**结论:**

Ⅰ期NSCLC解剖性肺叶切除术后复发转移以肺、脑、骨及纵隔淋巴结最常见，2年内与3年-5年复发转移风险相当，可以根据T分期调整2年内随访次数及随访内容。

## Abstract (en)

**Background and objective:**

Currently, there is no consensus on the follow-up strategy (follow-up time interval and content) of non-small cell lung cancer (NSCLC) in the world, and the relevant clinical evidence is also very limited. In this study, we aimed to summarize the recurrence/metastasis sites and timings of Stage Ⅰ NSCLC patients based on their follow-up data, aiming to provide a basis of follow-up time interval and content for this group of patients.

**Methods:**

We retrospectively analyzed the 416 Stage Ⅰ NSCLC patients that underwent continuous anatomic lobectomy between Jan. 2000 to Oct. 2013 in our prospective lung cancer database. According to the recurrence/metastasis sites and timings, the long term follow-up time interval and content were explored.

**Results:**

The 5-yr disease free survival (DFS) and overall survival (OS) in the whole group were 82.4% and 85.4%, respectively. There were 76 cases (18.3%) had recurrence/metastasis during follow-up, among which the most frequent site was pulmonary metastasis (21 cases, 5.0%), followed by brain metastasis (20 cases, 4.8%), bone metastasis (12 cases, 2.9%), and mediastinal lymph node metastasis (12 cases, 2.9%). Among the factors that could influence recurrence/metastasis, patients with pT2a suffered from a higher recurrence/metastasis rate compared to patients with pT1 (*P*=0.006), with 5-yr DFS being 73.8% and 87.3%, respectively (*P*=0.002), and the 5-yr OS being 77.7% and 90.3%, respectively (*P*=0.011).

**Conclusions:**

The commonest recurrence/metastasis sites of Stage Ⅰ NSCLC after anatomic lobectomy are lung, brain and mediastinal lymph nodes, the risk of recurrence/metastasis within 2 years were equal to that between 3 years and 5 years. The follow-up frequencies and content within 2 years could be adjusted according to T stages.

肺癌是全世界目前最常见的恶性肿瘤之一，而且发病率和死亡率仍在持续升高。非小细胞肺癌（nom-small cell lung cancer, NSCLC）是肺癌最常见的类型，其治疗有依赖于原发肿瘤（tumor, T）、淋巴结（node, N）和转移病灶（metastasis, M）的分期而决定^[1]^。早期NSCLC的主要治疗方法是以治愈为目的的外科治疗，然而并非所有早期患者均能被治愈，即便是Ⅰ期NSCLC术后仍有复发转移。因此，NSCLC患者术后需要终生随访，目的在于早期发现肿瘤的复发转移，进行适当的治疗，最终改善生存。但目前国际上对NSCLC的随访间隔时间及随访内容并未达成共识，各指南对于不同病理类型、不同分期NSCLC的随访策略一概而论、毫无差别，NSCLC术后2年内每3个月门诊随访，3年-5年为每半年门诊随访，5年后每年门诊随访^[1, 2]^。我们假设认为早期NSCLC随访内容过多、频率过高，会造成资源浪费甚至医源性伤害。因此，亟需相关的研究为临床提供可靠的参考数据。我科自2000年肺癌前瞻性数据库建立后，一直坚持定期门诊随访术后患者，积累了大量真实可靠的肺癌手术患者随访资料。本研究回顾性分析了经外科手术治疗的416例Ⅰ期NSCLC的随访资料，总结其复发转移的部位及时间，为制定该类患者的随访策略提供参考。

## 1 对象与方法

### 1.1 研究对象

选取我科肺癌前瞻性数据库中2000年1月-2013年10月单一医生组连续行解剖性肺叶切除手术的肺癌患者1, 290例作为筛选对象。入组标准：①肺原发性NSCLC者；②接受解剖性肺叶切除及系统性淋巴结清扫手术治疗者；③根据第8版国际抗癌联盟（Union for International Cancer Control, UICC）/美国癌症联合会（American Joint Committee on Cancer, AJCC）NSCLC TNM分期系统，术后病理分期为Ⅰ期者；④手术切缘阴性（R0切除）。排除标准：①既往恶性肿瘤病史者；②术前行新辅助治疗者；③围手术期死亡者（术后90天内）。根据入组及排除标准，最终共416例纳入研究。

### 1.2 诊断与治疗策略

术前分期检查包括胸部增强增强计算机断层扫描（computed tomography, CT）、全身正电子发射计算机断层显像（positron emission tomography/CT, PET/CT）（2008年以后）、头颅增强磁共振成像（magnetic resonance imaging, MRI）、全身骨扫描、颈腹部超声及气管镜检查，对于影像怀疑纵隔淋巴结阳性者行支气管内超声（endobroncheal ultrasonography, EBUS）穿刺或电视纵隔镜检查。肿瘤分期采用UICC和AJCC联合制定的第8版TNM分期系统^[3]^。手术以根治性为目的，全腔镜或开放手术行解剖性肺叶切除术加系统性淋巴结清扫。

### 1.3 随访方式

术后2年之内每3个月门诊复查一次，3年-5年每半年门诊复查一次，5年以后每年门诊复查一次。复查内容详细记录患者近期主诉及症状，检查内容包括胸部增强CT及颈腹部超声；头颅增强MRI/CT及全身骨扫描每半年至一年复查；全身PET/CT无症状时术后2年及5年复查；支气管镜及EBUS穿刺等检查根据患者症状及检查结果决定，不作为常规。本组患者均为门诊随访，截止日期为2018年1月15日或死亡，随访率98.7%；存活患者中位随访时间为71.2个月（12个月-208个月）。

### 1.4 研究指标

临床信息包括性别、年龄、吸烟史、术前合并症、手术信息、肿瘤病理及分期、肿瘤表皮生长因子受体（epithelial growth factor receptor, *EGFR*）基因突变情况及术后随访资料（末次随访状态、总生存时间、首次复发转移时间及部位）等。

### 1.5 统计学方法

统计分析采用SPSS 22.0分析（SPSS公司，芝加哥，伊利诺斯，美国）。临床因素组间复发转移比较采用卡方检验；单因素生存分析采用*Kaplan-Meier*法，用*Log-rank*进行显著性检验。统计结果均按照*P* < 0.05定义为差异具有统计学意义。

## 2 结果

### 2.1 基本资料

由1, 290例筛选出Ⅰ期NSCLC肺叶切除者416例，中位年龄62岁，18岁-82岁；其中男性214例（51.4%），女性202例（48.6%）；吸烟者166例（39.9%），非吸烟者250例（60.1%）；术前合并心肺疾病者148例（35.6%）；手术为全胸腔镜者192例（46.2%），传统开胸手术者224例（53.8%）；术后病理为腺癌者321例（77.2%），鳞癌者61例（14.7%），其他类型者34例（8.2%）；根据AJCC/UICC第8版TNM分期系统，术后病理分期为T1a者28例（6.7%），T1b者124例（29.8%），T1c者90例（21.6%），T2a者174例（41.8%）。全组共119例腺癌患者行肿瘤原发灶EGFR基因检测，阳性者90例（75.6%），阴性者29例（24.4%）（表 1）。

**1:** 患者基本资料及5年DFS Baseline characteristics and 5-yr DFS of patients

	*n*	Relapse and metastasis		*P*	5-yr DFS	*P*
		Yes	No			
Age (yr)				0.159		0.146
< 70	311	52 (16.7%)	259 (83.3%)		83.6%	
≥70	105	24 (22.9%)	81 (77.1%)		77.0%	
Gender				0.981		0.887
Male	214	39 (18.2%)	175 (81.8%)		82.2%	
Female	202	37 (18.3%)	165 (81.7%)		81.6%	
Smoking				0.862		0.974
Yes	166	31 (18.7%)	135 (81.3%)		80.0%	
No	250	45 (18.0%)	205 (82.0%)		82.5%	
Cardiopulmonary complications				0.065		0.031
Yes	148	34 (23.0%)	114 (77.0%)		75.9%	
No	268	42 (15.7%)	226 (84.3%)		85.3%	
Location				0.519		0.367
Right	249	43 (17.3%)	206 (82.7%)		83.9%	
Left	167	33 (19.8%)	134 (80.2%)		78.2%	
Surgery				0.072		0.288
VATS	192	28 (14.6%)	164 (85.4%)		84.2%	
Open	224	48 (21.4%)	176 (78.6%)		80.8%	
Histology				0.250		0.215
Ad	321	64 (19.9%)	257 (80.1%)		81.1%	
Sq	61	7 (11.5%)	54 (88.5%)		88.3%	
Others	34	5 (14.7%)	29 (85.3%)		84.8%	
pT stage				0.006		0.002
T1a	28	4 (14.3%)	24 (85.7%)		83.2%	
T1b	124	13 (10.5%)	111 (89.5%)		89.9%	
T1c	90	14 (15.6%)	76 (84.4%)		85.7%	
T2a	174	45 (25.9%)	129 (74.1%)		73.8%	
*EGFR* mutation				0.950		0.828
(+)	90	12 (13.3%)	78 (86.7%)		86.2%	
(-)	29	4 (13.8%)	25 (86.2%)		85.1%	
EGFR: epidermal growth factor receptor; VATS: video-assisted thoracic surgery; DFS: disease-free survival; Ad: adenocarcinoma; Sq: sqaumous cell carcinoma.						

### 2.2 全组患者术后生存分析及复发转移特征

全组患者5年DFS与OS分别为82.4%和85.4%。全组患者随访期间共有76例（18.3%）出现复发转移，复发转移部位中最常见依次为肺转移21例（5.0%），脑转移20例（4.8%），骨转移12例（2.9%），纵隔淋巴结转移12例（2.9%），肝转移6例（1.4%），胸膜转移3例（0.7%），皮下转移结节2例（0.5%）。术后每年新增复发转移情况见图 1。生存分析计算最常见复发转移部位2年、5年的累计复发率，肺转移者分别为2.3%（95%CI: 0.7%-3.9%）和4.8%（95%CI: 2.6%-7.0%）；脑转移者分别是2.5%（95%CI: 0.9%-4.1%）和6.0%（95%CI: 3.3%-8.7%）；骨转移者分别是1.5%（95%CI: 0.3%-2.7%）和3.3%（95%CI: 1.5%-5.1%）；纵隔淋巴结转移者分别是1.6%（95%CI: 0.4%-2.8%）和3.9%（95%CI: 1.2%-6.6%）。

**1:**
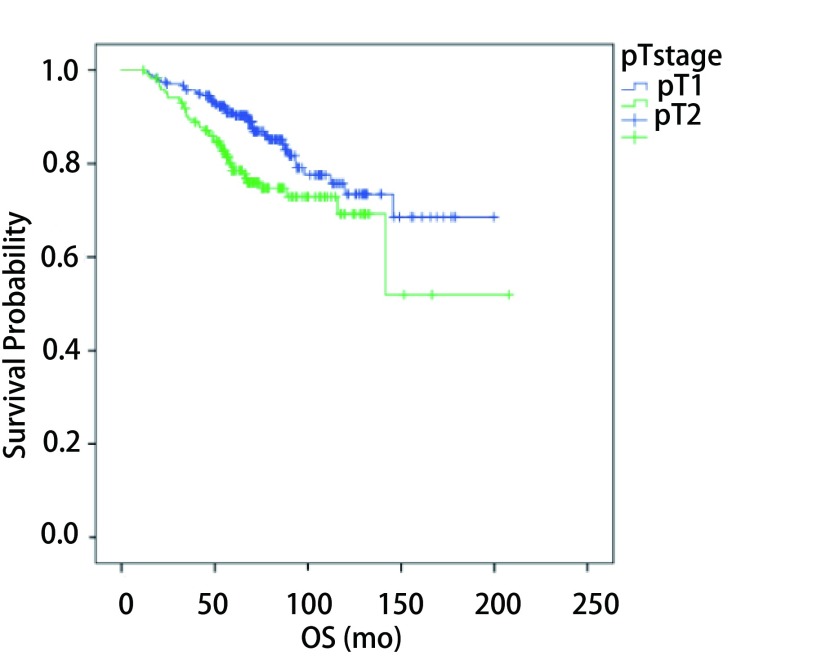
pT1期与pT2a期NSCLC术后患者OS *Kaplan-Meier*曲线 OS *Kaplan-Meier* curves for all 416 NSCLC patients with pT1 and pT2a after lobectomy. OS: overall survival; NSCLC : non-small cell lung cancer.

### 2.3 复发转移患者危险因素的单因素分析

研究在不同年龄、性别，是否吸烟、心肺合并症，不同肿瘤位置、手术方式、病理类型、pT分期及*EGFR*突变情况中，比较复发转移的情况及5年DFS。发现pT2a者复发转移率高于pT1者（*P*=0.006），5年DFS分别为73.8%和87.6%（*P*=0.002），5年OS分别为77.7%和90.3%（*P*=0.011）（图 2）；有术前心肺合并症者5年DFS较无心肺合并症者差（75.9% *vs* 85.3%，*P*=0.031）（表 1）。

**2:**
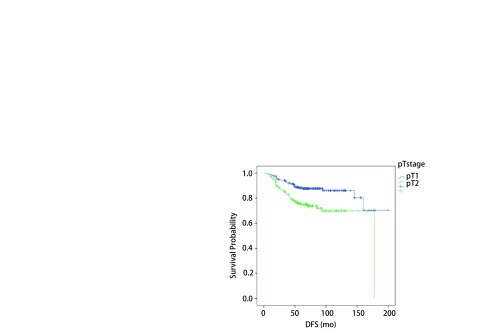
pT1期与pT2a期NSCLC术后患者DFS *Kaplan-Meier*曲线 DFS *Kaplan-Meier* curves for all 416 NSCLC patients with pT1 and pT2a after lobectomy. DFS: disease free survival.

### 2.4 患者症状与复发转移部位的关系

肺及纵隔淋巴结转移者绝大多数（85.7%和91.7%）就诊时无症状，以常规胸部增强CT检查发现；肝转移者大多数（66.7%）也是无症状就诊常规超声检查发现；胸膜、脑及骨转移的者大多数（66.7%、60.0%和58.3%）是因症状就诊检查发现；皮下转移结节均为患者自行发现后就诊（图 3）。

**3:**
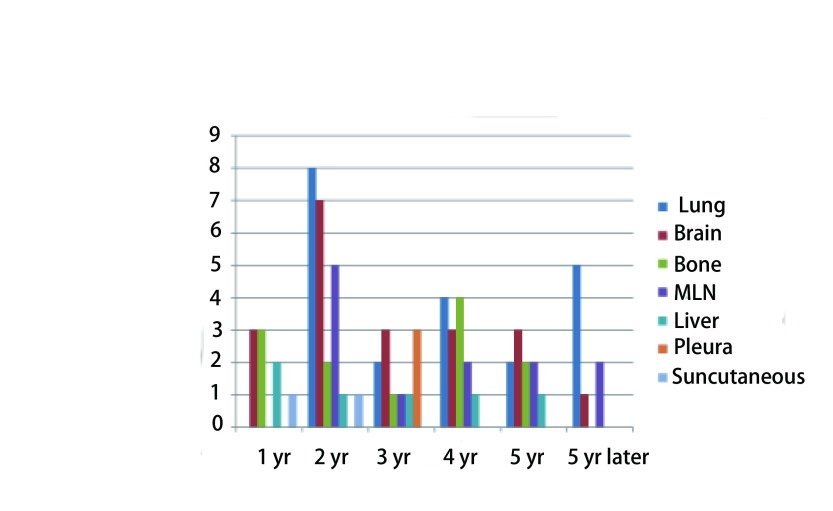
术后每年新增复发转移 New recurrence and metastasis every year after operation

## 3 讨论

外科是早期NSCLC的标准治疗，但术后仍然有复发转移，部分患者还会出现第二原发肿瘤^[4]^。因此，术后的随访监测显得尤为重要，目的是早期发现及治疗复发转移或第二原发肿瘤，最终改善生存。但目前国际上对于NSCLC的随访模式上并未达成共识，相关的临床证据也十分有限。究其原因，缺乏早期NSCLC术后长高质量随访导致此类患者术后复发转移模式不明确。

文献报道Ⅰ期NSCLC的5年DFS为84%-87%，本组患者5年DFS与OS分别为82.4%和85.4%，同以往报道近似^[5-7]^。Hung等^[8]^报道的756例手术治疗NSCLC，2年局部无复发率和无远处转移率分别为90.7%和82.1%，5年OS和复发率分别为57.3%和70.2%；T分期是影响总生存、整体复发率和远处转移率的主要因素，同时吸烟患者及非鳞癌患者的远处转移风险更高。但文章没有描述复发转移的具体部位和时间。我们全组患者随访期间仅有76例（18.3%）出现复发转移，低于文献报道，可能原因如下：①本组患者均为病理Ⅰ期NCSLC，未混杂临床Ⅰ期；②术前分期检查规范、统一，包括全身PET/CT检查，最大限度除外隐匿的远传转移；③单中心单一手术组病例，手术质量均一，全组患者均行系统淋巴结清扫，确切N0分期；④手术切除方式为标准肺叶切除，不包含亚肺叶切除病例。通常，肺癌术后常见复发转移部位依次为肺（包括支气管残端）、纵隔淋巴结、脑、骨、肝及肾上腺等，但早期肺癌由于大多数为周围性肺癌，解剖性肺叶切除术后极少出现局部支气管残端复发，有别于中心型肺癌复发转移部位。本组患者中复发转移部位中最常见依次为肺转移21例（5.0%），脑转移20例（4.8%），骨转移12例（2.9%），纵隔淋巴结转移12例（2.9%），而肝转移，胸膜转移在早期肺癌术后患者中属于罕见转移。转移时间上，从术后第一年就开始出现复发转移，绝大多数的复发转移出现在术后5年之内，2年内与3年-5年的复发转移率无明显差别；5年之后再复发转移率仅为1.9%。

对于NSCLC术后复发转移的预测因素，既往研究较多，所涉及的因素也很多，包括肿瘤TNM分期、围手术期治疗、肿瘤基因特性等，但最为大家接受的仍然是TNM分期^[9]^。本研究的对象均为Ⅰ期NSCLC，手术方式为统一的肺叶切除及系统性淋巴结清扫，消除了手术因素对预后产生的偏倚。在分析其他临床特征与预后关系时，我们发现T分期仍然是决定复发转移的最大因素。虽然有研究认为相对于其他病理类型，早期腺癌术后更容易出现复发转移，但我们研究发现，是否腺癌和*EGFR*基因是否突变均不会影响早期NSCLC的复发转移^[10]^。

肺癌手术治疗后的最初2年，是公认的复发高危期，因此大多学者均支持在这段时间给予患者相对高密度的随访，以期及早发现复发病灶。在临床实践中，由于缺乏针对早期NSCLC的随访指南，不同单位、不同部门甚至不同医生的随访策略差别迥异。我科目前的随访策略是：术后2年之内，每3个月进行随访；3年-5年期间，每6个月进行随访；5年以后，每年进行随访。检查内容包括：血液及生化室检查、胸部CT、颈部及腹部超声、头颅MRI（每6个月一次）、全身骨扫描或PET/CT（每年一次）。然而，本研究结果告诉我们，这种随访策略在早期NSCLC患者中并不恰当，可能存在随访过度。早期NSCLC术后复发转移率低，虽主要集中在术后5年之内，但2年内复发转移风险并不高于3年-5年，因此2年内复发间隔时间无需更频繁。而在随访内容方面，肺及纵隔淋巴结转移仍然是最常见转移，且少有症状，只能靠定期复查发现，因此术后前5年复查项目中仍应保留每年1次-2次胸部增强CT检查，5年后由于复发转移风险降低可每年1次；对于危害更大的脑、骨、肝脏等部位转移，大多数患者因相应的临床症状就诊发现，鉴于其同样的低发生率，建议头颅MRI及骨扫描检查可只在有症状时实施，5年后是否要加强随访值得考虑。在更理想的随访策略应当根据患者不同的复发和死亡风险进行分层，高风险者（如T2a者）给予相对积极和严格的随访，低风险者（如T1者）则相对保守和宽松。

本研究回顾性分析了Ⅰ期NSCLC经肺叶切除术后的长期生存结果，通过其复发转移特点探讨了对于此类患者的远期随访策略。但作为一项回顾性、单中心研究，不可避免的存在一些局限性和偏倚，应在外部数据库进行验证及对照研究。
